# Speicheldrüsenkarzinome – ein aktueller Überblick: Fortschritte in der molekularen Typisierung: Teil I

**DOI:** 10.1007/s00292-022-01123-y

**Published:** 2022-10-13

**Authors:** Niels J. Rupp, Sandra N. Freiberger

**Affiliations:** 1 Institut für Pathologie und Molekularpathologie, Universitätsspital Zürich, Universität Zürich, Zürich, Schweiz; 2 Schmelzbergstr. 12, 8091 Zürich, Schweiz

**Keywords:** Klassifikation, Morphologische und mikroskopische Befunde, Speicheldrüsenneoplasien, Genfusion, Mutation, Classification, Morphological and microscopic findings, Salivary gland neoplasms, Gene fusion, Mutation

## Abstract

In den letzten Jahren hat die Charakterisierung der Speicheldrüsenkarzinome einen großen Wandel durchlebt. Morphologisch definierte Entitäten konnten zu einem Großteil auch molekular mit einem oftmals distinkten Genotyp charakterisiert werden. Der erste Teil des Artikels gibt einen Überblick über die Fortschritte der molekularen Charakteristiken des Mukoepidermoidkarzinoms, adenoid-zystischen Karzinoms, Azinuszellkarzinoms, des sekretorischen und intraduktalen Karzinoms sowie des hyalinisierenden klarzelligen Karzinoms. Der molekulare Genotyp kann dabei insbesondere bei der Klassifizierung ungewöhnlicher morphologischer Varianten von großem Nutzen sein. Rekurrente *NTRK*- oder *RET*-Genfusionen können dabei nicht nur als diagnostisches Hilfsmittel, sondern auch für eine potenzielle gezielte Therapie genutzt werden.

## Abstract (en)

In recent years, the characterization of salivary gland tumors has undergone a major transformation. Morphologically defined entities could to a large extent also be characterized molecularly with an often distinct genotype. The first part of this article reviews the advances in the molecular characteristics of mucoepidermoid carcinoma, adenoid cystic carcinoma, acinic cell carcinoma, secretory carcinoma, intraductal carcinoma, and hyalinizing clear cell carcinoma. The molecular genotype can be particularly useful in classifying unusual morphologic variants. Recurrent *NTRK* or *RET* gene fusions can be used not only as a diagnostic tool but also for potential targeted therapy.

## Lernziele

Nach Lektüre dieses Beitrags …

- können Sie relevante molekularen Alterationen in Speicheldrüsenkarzinomen benennen;
- können Sie die immunhistochemischen Surrogatmarker der beschriebenen molekularen Alterationen sicherer evaluieren;
- kennen Sie die spezifischen molekularen Alterationen des Mukoepidermoidkarzinoms und des adenoid-zystischen Karzinoms;
- kennen Sie die molekularen Spektren zur Differenzialdiagnose des Azinuszellkarzinoms, sekretorischen Karzinoms und intraduktalen Karzinoms.

## Hintergrund

Speicheldrüsenkarzinome sind seltene Neoplasien, die aufgrund überlappender morphologischer Muster insbesondere an bioptischem und zytologischem Material herausfordernd sind. In den letzten Jahren konnten zahlreiche, oftmals spezifische molekulare Alterationen identifiziert werden, die insbesondere bei ungewöhnlichen morphologischen Varianten oder limitiertem Material diagnostisch sehr hilfreich sein können. Weiterhin sind **rekurrente Genfusionen**

rekurrente Genfusionen

nicht nur von diagnostischem Nutzen, sondern können potenziell auch gezielt therapeutisch angegangen werden. Diese Arbeit soll einen Überblick über den aktuellen Stand der morphomolekularen Typisierung von Speicheldrüsenkarzinomen geben.

## Fallbeispiel

Sie erhalten das laterale **Parotidektomiepräparat**

Parotidektomiepräparat

eines 58-jährigen Patienten. Histologisch zeigt sich eine relativ scharf umgrenzte Speicheldrüsenneoplasie von maximal 1,8 cm Durchmesser. Diese besteht aus ausschließlich zytologisch blanden, morphologisch onkozytär aufgebauten Zellen mit voluminösem eosinophilem Zytoplasma und rundlichen Kernen mit feinen Nukleolen. Stellenweise zeigen sich fibröse Bänder, die die Läsion durchziehen. Es finden sich praktisch keine Mitosefiguren, keine Nekrosen und keine Perineuralscheideninfiltration. Die breite **Differenzialdiagnose**

Differenzialdiagnose

umfasst benigne und „low grade“ maligne onkozytär differenzierte Speicheldrüsenneoplasien.

## Molekulare Eigenschaften etablierter Entitäten

### Mukoepidermoidkarzinom

**Figure 1:**
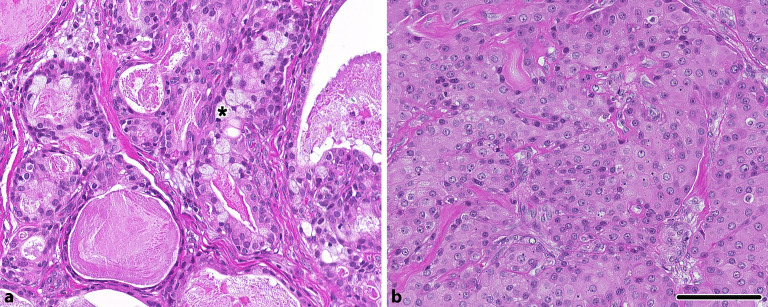

Das Mukoepidermoidkarzinom zeigt typischerweise ein triphasisches Muster unter Einschluss squamoider (epidermoider), intermediärer (zumeist heller) Zellen und Mukozyten (Abb. 1; [1]). Die klassischen Varianten lassen sich in der Regel ohne größere Schwierigkeiten diagnostizieren, jedoch besteht auch eine Reihe **ungewöhnlicher Varianten**

ungewöhnlicher Varianten.

Diese umfassen unter anderem die onkozytäre Variante (OMEC; Abb. 1), die sklerosierende, ziliierte oder auch Warthin-ähnliche Variante sowie das kürzlich beschriebene mukoazinäre Karzinom [2, 3, 4, 5]. Diese Varianten haben gemeinsam, dass sie häufig molekular eine Genfusion im *MAML2*-Gen, zumeist mit dem Fusionspartner *CRTC1 *oder seltener *CRTC3*, zeigen (Tab. 1) und die klassischen morphologischen Eigenschaften praktisch vollständig fehlen können. Diese Genfusionen sind hochspezifisch und im Kontext eines Speicheldrüsentumors molekular praktisch beweisend für das Spektrum der Mukoepidermoidkarzinome ([6]; cave: auch Adnextumoren der Haut wie das **Hidradenom**

Hidradenom

können identische Fusionen zeigen [7]). In einer kürzlich publizierten Studie konnten auch andere molekulare Alterationen, wie z. B. *HRAS*- oder *KRAS*-Mutationen detektiert werden, wobei diese ohne zusätzlichen Nachweis einer ***MAML2*-Umlagerung**

MAML2-Umlagerung

naturgemäß für die Entitätsdiagnose aufgrund fehlender Spezifität weniger hilfreich sind [8]. Das vorgestellte Fallbeispiel lässt sich durch den molekularen Nachweis einer *CRTC3*-*MAML2*-Genfusion einer onkozytären Variante eines Mukoepidermoidkarzinoms (OMEC) zuordnen.

**Table 1:** 

Entität	Molekulare Alteration	Quelle
Mukoepidermoidkarzinom	*CRTC1*-*MAML2*- und *CRTC3*-*MAML2*-Genfusion	[4]
Adenoid-zystisches Karzinom	*MYB-NFIB*-, *MYBL1*-*NFIB*-Genfusion; *NOTCH1*-Mutation	[9, 10]
Azinuszellkarzinom	*NR4A3*-Hochregulierung („enhancer hijacking“); *HTN3-MSANTD3*-Genfusion	[11, 12]
Sekretorisches Karzinom	*ETV6*-*NTRK3*-, *ETV6*-*RET*-, *ETV6*-*MET*-, *VIM*-*RET*-Genfusion	[13, 14, 15, 16]
Intraduktales Karzinom	*NCOA4*-*RET*-, *TRIM27*-*RET*-* und TRIM33*-*RET*-Genfusion; *BRAF*(V600E)-Mutation	[17, 18]
Hyalinisierendes klarzelliges Karzinom	*EWSR1*-*ATF1*-Genfusion, *EWSR1*-*CREM*-Genfusion	[19, 20]

#### Merke

Der Nachweis einer ***MAML2***-Genfusion kann die Diagnose eines Mukoepidermoidkarzinoms, insbesondere in ungewöhnlichen Varianten, molekular untermauern.

### Adenoid-zystisches Karzinom

**Figure 2:**
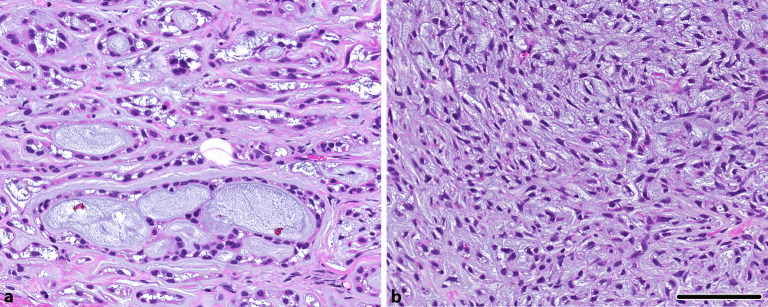

Das adenoid-zystische Karzinom zeigt klassischerweise einen **biphasischen Aufbau**

biphasischen Aufbau

mit peripheren myoepithelialen Zellen und zentralen duktal bzw. epithelial differenzierten Zellformationen. Das klassische Bild umfasst dabei eine tubuläre und kribriforme Differenzierung mit intra(pseudo)luminalem myxoidem Material (Abb. 2) neben **Inklusionen**

Inklusionen

hyalinisierter Basallamina. Bei ausgedehnter solider Komponente ist die Diagnostik erschwert, da die biphasische Differenzierung unter Umständen nicht mehr nachvollzogen werden kann. Außerdem können ungewöhnliche Varianten, wie eine sklerosierende Variante, eine spindelzellige Differenzierung (Abb. 2) oder das sog. metatypische adenoid-zystische Karzinom die eigentliche Tumorentität verschleiern [21, 22, 23]. Insbesondere Feinnadelpunktionen können diagnostisch sehr herausfordernd sein und eine Menge relevanter Differenzialdiagnosen umfassen. Hier kann der molekulare Nachweis einer *MYB*- oder ***MYBL1*-Genfusion**

MYBL1-Genfusion,

typischerweise mit dem Fusionspartner *NFIB*, diagnostisch beweisend sein [9]. Diese molekulare Alteration ist bisher in anderen Speicheldrüsentumoren nicht beschrieben. Eine weitere, seltenere Subgruppe mit häufiger solider Differenzierung zeigt pathogene *NOTCH1*-Mutationen, typischerweise ohne Nachweis einer *MYB*- oder *MYBL1*-Genfusion [10]. Gelegentlich kann ebenfalls der fehlende Nachweis einer immunhistochemischen Biphasizität verwirrend sein. Eine Negativität für p40 bzw. p63 ist dabei in etwa 10 % der Fälle beschrieben [24]. Ein weiterer relevanter Punkt ist die Diagnose einer „high grade“ **Transformation**

Transformation,

die typischerweise mit einer nuklearen Anaplasie, einer desmoplastischen Stromareaktion, Mikropapillen mit squamoider Differenzierung sowie einem deutlich erhöhten Ki-67-Proliferationsindex, Komedonekrosen und einem aberranten p53 Muster einhergeht [25]. Diese sollten von der **soliden Variante**

soliden Variante

(> 30 % solide Anteile gemäß Weltgesundheitsorganisation, WHO; [1]) abgegrenzt werden, da diese typischerweise einen nochmals aggressiveren biologischen Verlauf zeigen können.

#### Merke

Der Nachweis einer ***MYB***-***NFIB***- oder ***MYBL1***-***NFIB***-Genfusion kann die Diagnose eines adenoid-zystischen Karzinoms, insbesondere in ungewöhnlichen Varianten, molekular untermauern.

### Azinuszellkarzinom

Das Azinuszellkarzinom bereitet bei klassischer Morphologie in der Regel wenige diagnostische Schwierigkeiten, wobei in limitiertem Material, insbesondere in Feinnadelpunktionen der Zytologie, die Abgrenzung zum normalen azinären Speicheldrüsengewebe herausfordernd sein kann. In Einzelfällen kann auch die Abgrenzung zum sekretorischen Karzinom schwierig sein, was erklärt, dass vor Charakterisierung des sekretorischen Karzinoms diese typischerweise als Azinuszellkarzinome klassifiziert wurden [13]. Kürzlich konnte eine in hohem Maße rekurrente molekulare Alteration, das „enhancer hijacking“ des ***NR4A3*(*NOR‑1*)-Gens**

NR4A3(NOR-1)-Gens,

identifiziert werden [11]. Dabei kommt es zur Translokation von Enhancerabschnitten des SCPP-Genclusters vor das *NR4A3*-Gen, was konsekutiv zu einer starken **Überexpression**

**Figure 3:**
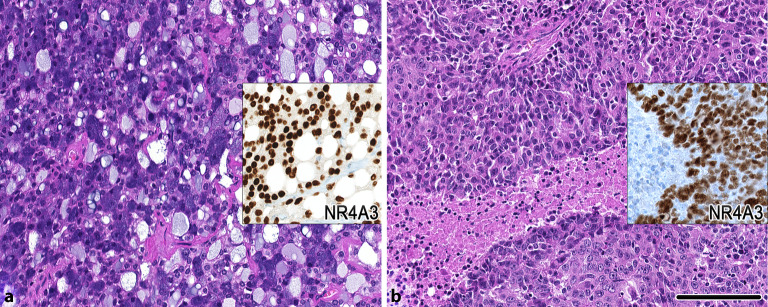

Überexpression

des *NR4A3*-Gens bzw. der entsprechenden mRNA und des Proteins führt. Dieses kann sowohl mittels quantitativer mRNA-Analyse sowie mittels spezifischer NR4A3-Immunhistochemie nachgewiesen werden ([22, 26]; Abb. 3). Die Fluoreszenz-in-situ-Untersuchung (FISH) von *NR4A3* kann ein positives **Break-apart-Muster**

Break-apart-Muster

zeigen, sofern die Region des Bruchpunkts über das Sondendesign abgedeckt ist [11]. Am besten diagnostisch verwertbar ist jedoch die „downstream“ gelegene NR4A3-Überexpression: Die Immunhistochemie kann auch erfolgreich an Zellblockmaterial von Feinnadelpunktionen mit sehr hoher Sensitivität und Spezifität (≥ 90 %) durchgeführt werden [27]. Auch Azinuszellkarzinome mit „high grade“ Transformationen, die die typische Morphologie verlieren können, sind oftmals mittels NR4A3-Expression zu überführen (Abb. 3). In sehr seltenen Fällen sind *HTN3*-*MSANTD3*-Genfusionen beschrieben [12].

#### Merke

Eine positive immunhistochemische Expression von NR4A3 kann die Diagnose eines Azinuszellkarzinoms, insbesondere bei ungewöhnlicher Morphologie, untermauern.

### Sekretorisches Karzinom

**Figure 4:**
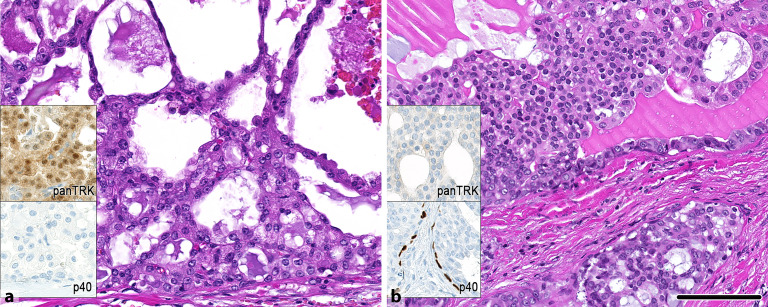

Das sekretorische Karzinom ist mittlerweile gut charakterisiert und kann verschiedene morphologische Varianten zeigen, darunter z. B. die makrozystische Variante [13, 28]. Morphologisch gemeinsam haben sekretorische Karzinome typischerweise ein voluminöses Zytoplasma mit „hobnail“-artigen Kernen und blasigen Sekretionen (Abb. 4). Die Immunhistochemie zeigt charakteristisch eine Positivität für **Mammaglobin**

Mammaglobin,

S‑100 und MUC4 [29]. In den meisten Fällen (> 90 %) findet sich molekular eine *ETV6*–*NTRK3*-Genfusion, in selteneren Fällen sind auch *ETV6*-*RET-, ETV6*-*MET-* und *VIM*–*RET*-Genfusionen beschrieben [14, 15, 16]. Typischerweise zeigen die *NTRK3*-translozierten sekretorischen Karzinome eine immunhistochemische, nukleäre Expression von panTRK (Abb. 4), sodass diese in Zusammenschau mit der Morphologie und dem übrigen Immunphänotyp diagnostisch verwendet werden kann [30].

#### Merke

Eine nukleäre panTRK-Expression kann auf die für sekretorische Karzinome typische ***ETV6-NTRK3***-Genfusion hinweisen.

### Intraduktales Karzinom

Das sog. intraduktale Karzinom (IDC) ist neben dem Azinuszellkarzinom die wichtigste Differenzialdiagnose zum sekretorischen Karzinom. Je nach Subtyp des IDC teilt es sich das Immunprofil (S‑100- und Mammaglobinexpression) mit dem sekretorischen Karzinom. Es zeigt jedoch zusätzlich klassischerweise eine zweite, myoepithelial differenzierte Zellpopulation (Abb. 4), ist negativ für MUC4 bzw. panTRK und weist je nach morphologischem Subtyp ein molekular distinktes Spektrum auf (u. a. *NCOA4*-*RET*-, *TRIM27*-*RET*-*, TRIM33*-*RET*-*Genfusion, BRAF*(V600E)-Mutation; [17, 18]).

### Hyalinisierendes klarzelliges Karzinom

Das hyalinisierende klarzellige Karzinom (HCCC) ist eine in der klassischen Form langsam wachsende Entität und findet sich üblicherweise in der oralen Mukosa. Es weist einen hyalinisierten Hintergrund mit trabekulären und strangförmigen Tumorzellproliferationen mit eosinophil- bis klarzelligem, optisch leerem Zytoplasma auf [31]. Immunhistochemisch exprimieren die Zellen typischerweise **squamoide Marker**

squamoide Marker,

wie p63, p40 oder CK5/6 [1], was gleichzeitig als Abgrenzung zu einer Metastase eines klarzelligen Nierenzellkarzinoms hilfreich sein kann. Molekular finden sich rekurrente Genfusionen im *EWSR1*-Gen, am häufigsten *EWSR1*-*ATF1*-Translokationen [19], seltener *EWSR1*-*CREM*-Translokationen [20]. Das HCCC zeigt ein überlappendes morphologisches und molekulares Profil mit dem klarzelligen odontogenen Karzinom (CCOC) des Kiefers [31].

## Fazit für die Praxis

- Speicheldrüsenkarzinome zeigen häufig typische molekulare Alterationen.
- Genfusionen zeigen dabei in der Regel eine hohe Spezifität.
- Molekulare Alterationen können im Kontext mit der Morphologie zur Diagnoseunterstützung bzw. Entitätseinordnung verwendet werden.
- Immunhistochemische Surrogatmarker, wie NR4A3 und panTRK, können diagnostisch hilfreich sein.
- Die *NTRK*- oder *RET*-Genfusionen können als potenzielles molekulares Ziel dienen.

## CME-Fragebogen

Welche ist die typische und diagnostische molekulare Alteration in Mukoepidermoidkarzinomen der Speicheldrüse?

*CRTC1*-*MAML2*-Genfusionen

*MYB*-*NFIB*-Genfusionen

*MYBL1*-*NFIB*-Genfusionen

*EWSR1*-*ATF1*-Genfusionen

*HTN3*-*MSANTD3*-Genfusionen

Welche sind typische und diagnostische molekulare Alterationen in adenoid-zystischen Karzinomen?

*HRAS*-Punktmutationen

*MYB*-*NFIB*- und *MYBL1*-*NFIB*-Genfusionen

*ETV6*-*RET*-Genfusionen

*BRAF*-Punktmutationen

*HTN3*-*MSANTD3*-Genfusionen

Mit welchem molekularem Mechanismus wird die NR4A3-Expression in Azinuszellkarzinomen am besten erklärt?

Punktmutation von Enhancer-Elementen

Translokation von Enhancer-Elementen

Genamplifikation von Enhancer-Elementen

Fehlender proteolytischer Abbau von Enhancer-Elementen

Deletion von Enhancer-Elementen

Sie sehen eine überwiegend solide und fokal kribriforme, biphasische maligne Neoplasie in der Parotis. Molekular findet sich eine pathogene *NOTCH1*-Mutation. Zu welcher Entität passt diese Konstellation am besten?

Adenoid-zystisches Karzinom

Sekretorisches Karzinom

Intraduktales Karzinom

Mukoepidermoidkarzinom

Azinuszellkarzinom

Mit welcher Methodik ist die typische molekulare Alteration im Azinuszellkarzinom am besten zu erkennen?

*NR4A3*-Fluoreszenz-in-situ-Hybridisierung

*NR4A3*-DNA-Sequenzierung

*NR4A3*-Immunhistochemie

*NR4A3*- Fragmentlängenanalyse

*NR4A3*-Chromogen-in-situ-Hybridisierung

Sie sehen ein partiell zystisches Karzinom der Speicheldrüsen mit voluminösem Zytoplasma und fokalen blasigen Sekretionen. Welche immunhistochemischen Untersuchungen helfen Ihnen vor allem in der Differenzialdiagnose zwischen sekretorischem und intraduktalem Karzinom?

p40 und MUC4

p40 und NR4A3

RAS^Q61R^ und NR4A3

HER2/neu und panTRK

Androgenrezeptor und HER2/neu

Gegen welche molekularen Alterationen besteht die grundsätzliche Möglichkeit einer gezielten Therapie?

*RET*- und *NTRK*-Genfusionen

*MYB*- und *MYBL1*-Genfusionen

*MAML2*- und *MYB*-Genfusionen

*NR4A3*- und *ETV6*-Genfusionen

*EWSR1*- und *HTN3*-Genfusionen

Sie haben in der oralen Mukosa ein eosinophiles bis klarzelliges Karzinom mit *EWSR1*-*ATF1*-Genfusion diagnostiziert. Welche der Aussagen trifft zu?

Hyalinisierende klarzellige Karzinome sind typischerweise hochaggressiv

Eine klinisch-radiologische Korrelation ist anzuraten

Hyalinisierende klarzellige Karzinome zeigen typischerweise eine zusätzliche *BRAF*(V600E)-Mutation

Seltenere Genfusionen im hyalinisierenden klarzelligen Karzinom umfassen *EWSR1* und *FLI1*

Das klarzellige Nierenzellkarzinom als Differenzialdiagnose zeigt häufig ebenfalls *EWSR1*-*ATF*1‑Genfusionen

Etwa wie viel Prozent der sekretorischen Karzinome zeigen eine *ETV6*-*NTRK3*-Genfusion?

< 10 %

Ca. 20–30 %

Ca. 40–50 %

Ca. 60–70 %

> 90 %

Welche beiden immunhistochemischen Surrogatmarker können Ihnen insbesondere in der Differenzialdiagnose zwischen einem sekretorischen Karzinom und einem Azinuszellkarzinom helfen?

Androgenrezeptor und NR4A3

HER2/neu und panTRK

panTRK und NR4A3

Androgenrezeptor und HER2/neu

RAS^Q61R^ und NR4A3
